# Prognostic value of a simple distance index derived from PET maximum intensity projection

**DOI:** 10.3389/fmed.2025.1565525

**Published:** 2025-07-14

**Authors:** Isaac Kargar Samani, Olivier Gheysens, Maxime Regnier, Alix Collard, Marc André, Eric Van Den Neste, Thierry Vander Borght

**Affiliations:** ^1^Department of Nuclear Medicine, CHU UCL Namur, Godinne Site, UCLouvain, Namur, Belgium; ^2^Department of Nuclear Medicine, Cliniques universitaires Saint-Luc, UCLouvain, Woluwe-Saint-Lambert, Belgium; ^3^Namur Research Institute for Life Sciences, UNamur, Namur, Belgium; ^4^Statistical Support Unit, Cliniques universitaires Saint-Luc, UCLouvain, Woluwe-Saint-Lambert, Belgium; ^5^Department of Hematology, CHU UCL Namur, Godinne site, UCLouvain, Namur, Belgium; ^6^Department of Hematology, Cliniques universitaires Saint-Luc, UCLouvain, Woluwe-Saint-Lambert, Belgium

**Keywords:** [^18^F]FDG PET/CT, oncology, lymphoma, DLBCL, dissemination

## Abstract

**Introduction:**

Dissemination indices derived from [^18^F]FDG PET/CT, such as Dmax, Dmax_bulk_, SPREAD_bulk_, SPREAD_patient_, and Dmax_Vox_ are validated prognostic biomarkers in diffuse large B-cell lymphoma. We introduce Dmax_VoxMIP_, the distance between the outermost voxels of the two most distant lesions on a 2D maximum intensity projection image, which is easy and straightforward to obtain. Our goal is to evaluate Dmax_VoxMIP_’s prognostic value compared to other features for easier clinical application.

**Methods:**

Metabolic tumor volume and dissemination indices were obtained from LIFEx, while Dmax_VoxMIP_ was obtained from Telemis and OsiriX.

**Results:**

Dmax_VoxMIP_ was not significantly higher in deceased than in living patients. However, patients with Dmax_VoxMIP_ values above the derived cutoff showed a shorter survival. By combining MTV and Dmax_VoxMIP_, we obtained 3 risk groups for OS and PFS.

**Discussion:**

Dmax_VoxMIP_ could advantageously replace other dissemination parameters as a prognostic index in patients with DLBCL.

## Introduction

Diffuse large B-cell lymphoma (DLBCL) is the largest subtype of malignant lymphoma characterized by a large diversity of presentations, treatments and outcomes due to multiple histologic subtypes, genetic abnormalities, and origin of cells. In routine clinical practice, several prognostic models such as the international prognostic index (IPI) or the national comprehensive cancer network (NCCN)-IPI, are valuable to predict overall survival (OS) and progression free survival (PFS). DLBCL is the most common and aggressive histological subtype of non-Hodgkin’s lymphoma (1) with around 30% of patients experiencing refractory disease or relapse (2). Hence, it is crucial to have precise prognostic markers to recognize patients with an elevated risk of advancing or experiencing a recurrence as they could potentially gain from an early transition to innovative therapies designed to enhance their prognosis.

 In the past decade, biomarkers derived from fluorine-18 fluorodeoxyglucose positron emission tomography ([^18^F]FDG PET), such as metabolic tumor volume (MTV)—a quantitative parameter representing the total volume of tumor tissue exhibiting radiotracer uptake above a defined threshold and therefore reflecting the metabolically active portion of the tumor -, have been proven to improve risk classification of DLBCL patients because they better reflect tumor burden compared with the Ann Arbor stage or the notion of bulky disease. Large prospective studies have demonstrated the superiority of MTV as prognostic feature over the commonly used prognostic indices (3, 4).

**FIGURE 1:**
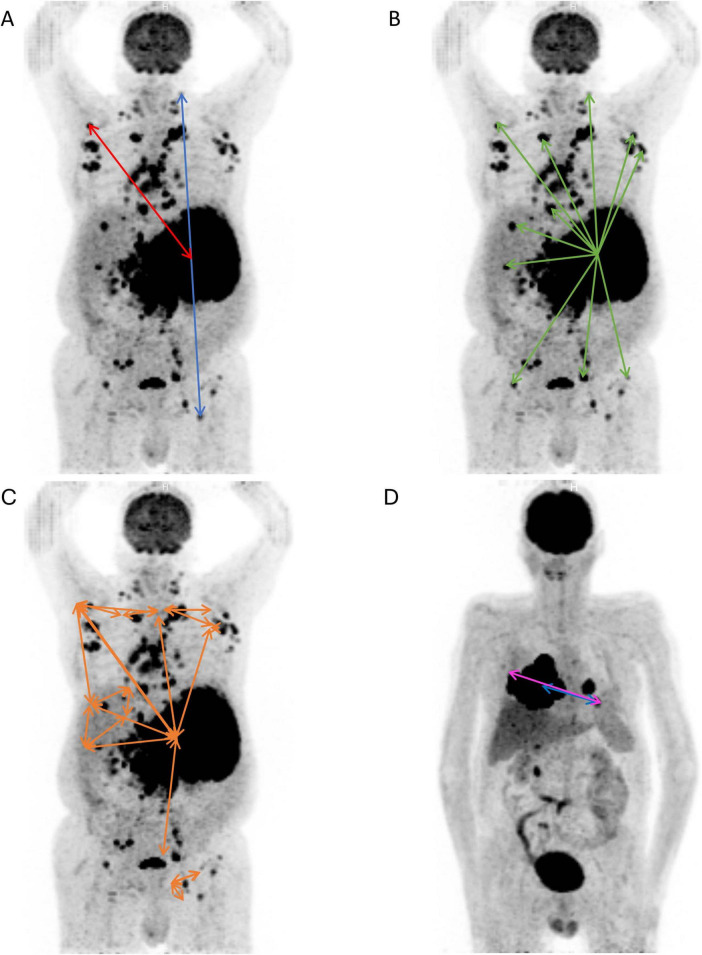
Representation of the different dissemination parameters used in this study. **(A)** Dmax_bulk_ (red arrow) is the distance between the metabolic center of the largest lesion and the most distant lesion from it (6, 7). Dmax (blue arrow) is the distance between the metabolic center of the two most distant lesions (6, 7). **(B)** SPREAD_bulk_ (green arrows) is the sum of the distances from the metabolic center of the largest lesion to the center of every other lesion (6, 7). **(C)** SPREAD_patient_ (orange arrows) is the largest value, over all lesions, of the sum of the distances from a lesion to all the others (6, 7). **(D**) Representative picture illustrating the difference between Dmax (blue arrow) and Dmax_Vox_ (pink arrow) which is the distance between the outermost voxels of the two most distant lesions, and cannot be differentiated from Dmax_VoxMIP_ on a 2D image. For illustration purposes, only a selection of arrows is shown; these do not represent all distances measured.

More recently, lesion dissemination indices derived from [^18^F]FDG PET have been introduced because MTV measurements do not reflect the heterogeneity of the spatial distribution of lesions often encountered in DLBCL patients. Several lesion dissemination parameters such as Dmax, Dmax_bulk_, SPREAD_bulk_, SPREAD_patient_ and Dmax_Vox_ [also called SDmax_Euc_Vox or SDmax_Vox when normalized to the body surface area (BSA) (5)] have been validated as prognostic biomarkers in diffuse large B-cell lymphoma (DLBCL) (5–9). The distance measured for Dmax and Dmax_Vox_ is the euclidean distance. The definition of several lesion dissemination indices is graphically represented in Figure 1.

Dmax intuitively reflects the spatial extent of the disease and is an easily measurable dimensional feature that is less affected by acquisition or reconstruction parameters than other PET metabolic indices. In addition, its automatic measurement eliminates operator dependency, and several software tools are nowadays available to perform an accurate and reproducible analysis. Therefore, Dmax can potentially better reflect the tumor’s capacity to disseminate, endowing it with stronger prognostic power than the traditional Ann Arbor stage (10). However, one of its limitations is that it cannot be used in patients with a single lesion. Secondly, it remains unclear whether this feature is dependent on patient height and/or body composition, although several studies showed a better performance of Dmax when normalized to BSA (5, 8). To overcome some of the limitations of ‘standard’ Dmax values, the concept of SDmax_Vox has been proposed by Cottereau et al. (5) and the use of 2D MIP was validated in the study of Girum et al. (11). In the latter study, Dmax was measured on sagittal and coronal MIPs using an artificial intelligence (AI) algorithm. Therefore, the purpose of this study was to investigate the prognostic value of a novel index, Dmax_VoxMIP_, defined as the distance between the outermost voxels of the two most distant lesions or of a single lesion on a 2D MIP image in the coronal view in comparison to other established dissemination features.

Compared to all the other dissemination parameters, Dmax_VoxMIP_ requires only a simple medical image viewing program (Telemis, OsiriX) and is therefore very easy to obtain. Our objective here is to demonstrate the prognostic value of Dmax_VoxMIP_ in relation to the other dissemination features as it would allow for a simpler use of these in everyday practice. Furthermore, the use of such simplified indices may be particularly valuable in settings where access to advanced or commercial image processing tools is limited, thereby enhancing the applicability of prognostic markers across a broader range of clinical environments.

## Materials and methods

### Patients

We performed a bi-centric retrospective study including all consecutive adult patients with a *de novo* diagnosis of DLBCL between 2008 and 2017 who were treated either at the Centre Hospitalier Universitaire UCLouvain Namur or at the Cliniques universitaires Saint-Luc (Belgium). All patients underwent a baseline fluorine-18 fluorodeoxyglucose positron emission tomography/computed tomography ([^18^F]FDG PET/CT) before receiving any treatment, and were subsequently treated with R-CHOP or R-CHOP like chemotherapy. All patients had at least a 5-year follow-up. Of note, patients who died before 5 years were included. We excluded patients who underwent a stand-alone PET without CT, patients with a primary central nervous system lymphoma or patients with a [^18^F]FDG PET/CT exam divided into an “ear-nose-throat” acquisition and a “rest of body” acquisition each containing lesions, precluding accurate measurement of dissemination indices. At last, we excluded patients who were diagnosed less than 5 years prior to the study or of whom we lost touch within 5 years. This study was approved by the institutional review board and patient consent was waived because of the retrospective nature and analysis of anonymized data.

### [^18^F]FDG PET/CT acquisition and analysis

[^18^F]FDG PET/CT images were obtained using a Gemini TOF-16 PET-CT camera (Philips Medical Systems). All our patients fasted for at least 6 h before FDG injection, with blood glucose levels systematically controlled at < 175 mg/dl. Injected activity ranged from 203 to 363 MBq. Iterative image reconstruction was performed using the Ordered-Subsets Expectation Maximization (OSEM) algorithm with 33 subsets and 3 iterations and with time-of-flight (TOF) information incorporated. A voxel size of 4 mm was employed throughout the process, with no additional smoothing applied to the images.

All dissemination and metabolic parameters, except Dmax_VoxMIP_, were obtained using LIFEx (12). Dmax_VoxMIP_ was measured manually on the 2D MIP image in the coronal view using the Telemis and OsiriX DICOM Viewer programs. To reduce potential bias, these measurements were carried out independently by observers who were blinded to clinical outcomes. For MTV measurement, an adaptive SUV-based threshold method (Nestle) was used as previously reported (13–16).

For the sake of simplicity, we chose to omit normalization of the dissemination indices to body surface area, since this yielded fairly similar statistical results.

### Statistical analysis

Statistical analysis was performed using The R Project for Statistical Computing 4.2.0 and SAS 9.4. Variables were summarized by their median and interquartile range (IQR) and compared between groups using the Wilcoxon-Mann-Whitney test. Comparisons were made between patients alive at 5 years and those deceased at 5 years, as well as between patients with and without an event at 5 years of follow-up. PFS events were defined as recurrence, progression, or death from any cause. To determine whether imaging variables improve the 5-year survival prediction of the international prognostic index (IPI) score, we used the Wald Chi-Squared test. The method of Contal and O’Quigley (17) based on logrank statistics was employed to categorize patients into “High” or “Low” risk groups for overall and progression-free survival based on each of the imaging variables. Cox proportional hazards model was used to determine the hazard ratio (HR) and its 95% confidence interval (CI95) between the high and low categories and then overall and progression-free survival curves were drawn using the Kaplan-Meier approach. Statistical significance was established for a *p*-value of < 0.05. In order to assess the reproducibility of Dmax_VoxMIP_, two more observers each analyzed 20 different patients on both Telemis and OsiriX. Bland-Altman plots were created to evaluate the agreement between the measurements.

## Results

### Patient characteristics

The clinical characteristics of the 104 patients included are shown in Table 1. Most patients had an advanced disease stage (73/103, 71%) and the majority were > 60 years old (66%). Median follow-up was 68.6 months (range: 0.53–166.2).

**TABLE 1:** Patients characteristics.

	N
**Age**	
Median	68 (18–89)
< 60 years old	35 (34%)
≥ 60 years old	69 (66%)
**Ann Arbor Stage**	
1	7 (7%)
2	24 (23%)
3	12 (12%)
4	61 (59%)
**ECOG performance status**	
0	24 (23%)
1	45 (43%)
2	18 (17%)
3	12 (12%)
4	5 (5%)
**Score IPI**	
0	3 (3%)
1, 2	42 (40%)
3, 4, 5	59 (57%)

At the time of data collection and last follow-up, 50 patients had died from any cause. Of these, 35 patients had died within 5 years of lymphoma diagnosis and 29/35 died of lymphoma. Of the 15 patients who died after a 5-year follow-up period, 2 deaths were lymphoma-related. At time of data collection, 54 patients had a PFS event (relapse, progression, or death of any cause), including 39 within the 5-year follow-up. The 5-year overall survival (5-y OS) and 5-year progression free survival (5-y PFS) were 66 and 63% respectively.

### PET indices

The Bland-Altman plot in Supplementary Figure 1 (comparing Dmax_VoxMIP_ measurements on the same PET by the same observer) shows good repeatability.

**FIGURE 2:**
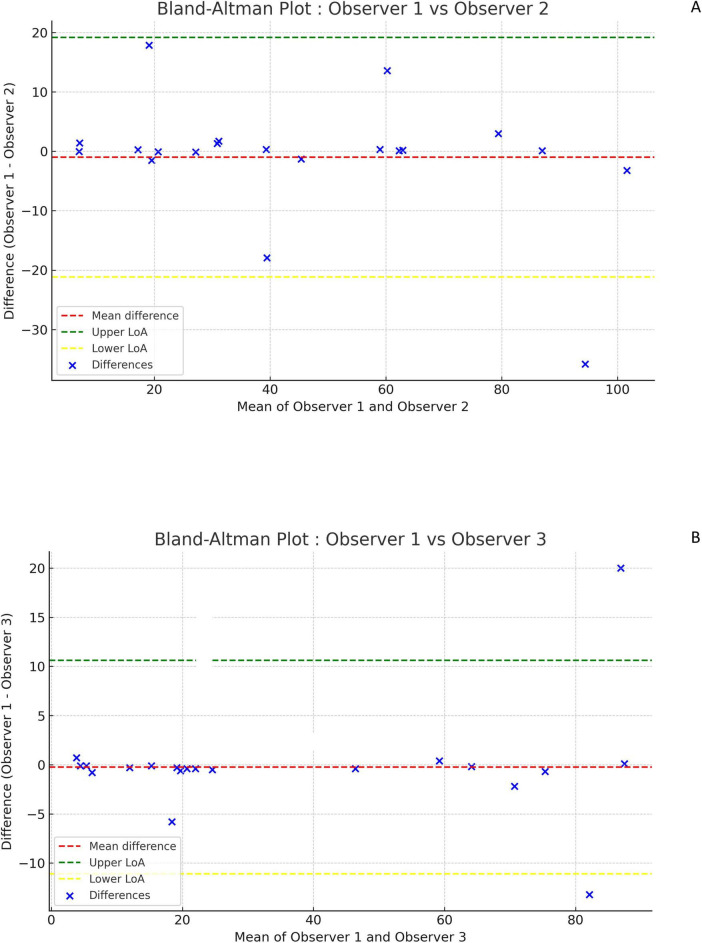
Bland-Altman plots comparing Dmax_VoxMIP_ measurements between observers. Difference between measurements of observer 1 and 2 versus their mean **(A)**. Difference between measurements of observer 1 and 3 versus their mean **(B)**.

**FIGURE 3:**
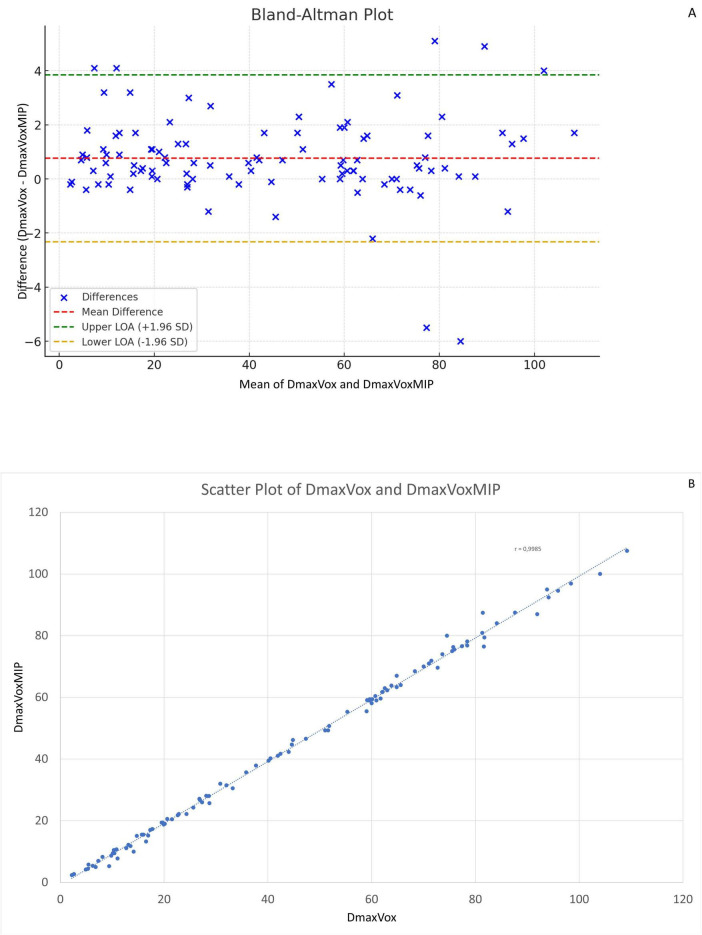
Bland-Altman plots comparing Dmax_Vox_ and Dmax_VoxMIP_ **(A)** and scatterplot showing the correlation between Dmax_Vox_ and Dmax_VoxMIP_ **(B)**. Difference between measurements of Dmax_Vox_ and Dmax_VoxMIP_ versus their mean **(A).**

The Bland-Altman plots in Figure 2A (comparing Dmax_VoxMIP_ measurements between observer 1 and 2) and Figure 2B (between observer 1 and 3) and in Supplementary Figure 2 (between observer 2 and 3) show only small differences between observers and no systematic bias. Likewise, the Bland-Altman plot comparing Dmax_Vox_ and Dmax_VoxMIP_ in Figure 3A shows small differences and no systematic bias. The scatterplot (Figure 3B) shows a strong positive correlation between Dmax_Vox_ and Dmax_VoxMIP_ with R of 0.9985.

MTV was significantly higher in patients who died within 5 years compared to those alive at 5-year follow-up (*p* = 0.031). In contrast, no significant difference was found between groups for any of the dissemination features (Table 2).

**TABLE 2:** Comparison of variables between groups alive at 5 years and deceased at 5 years.

Variables	Alive at 5 years (*n* = 69)		Deceased at 5 years (*n* = 35)		*p*-value
	Median [IQR]	Range	Median [IQR]	Range	
MTV (mL)	298.00 [60.00, 1148.00]	2; 8368	534.00 [266.50, 1792.00]	19; 5509	0.031
Dmax (cm)	30.20 [8.10, 60.60]	0; 93.4	47.70 [20.50, 66.60]	3.9; 101.2	0.151
Dmax_Vox_ (cm)	37.70 [14.70, 62.50]	2.2; 98.4	51.00 [24.70, 72.55]	9.4; 109.2	0.106
Dmax_VoxMIP_ (cm)	37.90 [15.10, 62.30]	2.4; 96.9	49.30 [23.75, 72.95]	5.3; 107.5	0.107
Dmax_bulk_ (cm)	27.10 [8.10, 43.80]	0; 72.7	33.40 [14.10, 45.95]	3.9; 83.7	0.176
SPREAD_bulk_ (cm)	67.90 [13.00, 300.00]	0; 2227	197.00 [56.65, 478.50]	3.9; 2044	0.104
SPREAD_patient_ (cm)	291.00 [31.10, 6994.00]	0; 145052	1452.00 [313.50, 10341.50]	7.8; 158218	0.097

Patients who experienced an event within 5 years had significantly higher MTV values compared to those who did not (*p* = 0.027). Similarly, Dmax_Vox_ (*p* = 0.048), SPREAD_bulk_ (*p* = 0.044) and SPREAD_patient_ (*p* = 0.037) showed significant differences between the two groups. Dmax_VoxMIP_ was higher in the event group than in the non-event group, but not significantly so (*p* = 0.052) (Table 3).

**TABLE 3:** Comparison of variables between groups with and without event at 5 years of follow-up.

Variables	No PFS event at 5 years (*n* = 65)		PFS event at 5 years (*n* = 39)		*p*-value
	Median [IQR]	Range	Median [IQR]	Range	
MTV (ml)	284.00 [60.00, 1101.00]	2; 8368	534.00 [240.50, 1759.00]	19; 5509	0.027
Dmax (cm)	30.20 [8.10, 60.10]	0; 93.4	53.30 [19.90, 67.40]	3.9; 101.2	0.069
Dmax_Vox_ (cm)	35.80 [14.70, 62.10]	2.2; 98.4	59.10 [22.60, 75.50]	9.4; 109.2	0.048
Dmax_VoxMIP_ (cm)	35.70 [15.10, 61.80]	2.4; 96.9	58.1 [21.80, 75.00]	5.3; 107.5	0.052
Dmax_bulk_ (cm)	26.80 [8.10, 43.40]	0; 72.7	33.40 [13.80, 46.60]	3.9; 83.7	0.118
SPREAD_bulk_ (cm)	46.50 [13.00, 290.00]	0; 2227	198.00 [50.30, 510.00]	3.9; 2044	0.044
SPREAD_patient_ (cm)	280.00 [32.10, 6591.00]	0; 139349	2013.00 [261.00, 12173.00]	7.8; 158218	0.037

As illustrated in Table 4, adding PET metabolic or dissemination indices to the IPI variable did not significantly improve the discrimination between deceased and surviving patients, neither between patients free of disease and patients with a PFS event at 5-year follow-up.

**TABLE 4:** Hazard ratios for OS and PFS for imaging variables added to a survival model already containing the IPI.

	5-y OS				5-y PFS			
Variables	HR	Lower limit	Upper limit	^¶^ *p*-value	HR	Lower limit	Upper limit	^¶^ *p*-value
Dmax	1.395	0.396	4.915	0.6046	1.555	0.758	3.190	0.2288
Dmax_Vox_	1.696	0.409	7.037	0.4669	1.489	0.732	3.028	0.2717
Dmax_VoxMIP_	1.295	0.416	4.032	0.6559	1.489	0.732	3.028	0.2717
Dmax_bulk_	1.543	0.441	5.400	0.4976	1.283	0.413	3.989	0.6669
SPREAD_bulk_	1.427	0.541	3.767	0.4726	1.522	0.617	3.752	0.3621
SPREAD_patient_	1.249	0.478	3.266	0.6497	1.365	0.558	3.340	0.4950
MTV	1.290	0.516	3.229	0.5861	1.447	0.618	3.384	0.3944

^¶^ *p*-value of the Wald Chi-square test for the addition of the imaging variable to the survival model already containing IPI.

Table 5 shows the hazard ratio with 95% CI for the 5-year OS and PFS for each individual PET-derived variable considering the group “Low” as a reference. All imaging features provided significant prognostic information with regard to 5-y OS and 5-y PFS.

**TABLE 5:** PET parameters associated with OS and PFS in log-rank cox tests.

	5-y OS					5-y PFS				
Variables	Cutoff	Survival rate “High”	Survival rate “Low”	HR (95%CI)	*p*-value	Cutoff	Survival rate “High”	Survival rate “Low”	HR (95%CI)	*p*-value
Dmax	10.1 cm	61% (31/79)	84% (4/25)	2.97 (1.05–8.43)	0.0313	61.8 cm	44% (15/27)	69% (24/77)	2.29 (1.20–4.37)	0.0097
Dmax_Vox_	16 cm	61% (32/82)	86% (3/22)	3.50 (1.07–11.45)	0.0268	63.8 cm	47% (16/30)	69% (23/74)	2.16 (1.14–4.09)	0.0156
Dmax_VoxMIP_	19 cm	61% (30/76)	82% (5/28)	2.70 (1.05–6.96)	0.0323	63.3 cm	47% (16/30)	69% (23/74)	2.16 (1.14–4.09)	0.0156
Dmax_bulk_	10.1 cm	60% (31/78)	85% (4/26)	3.15 (1.11–8.93)	0.0227	10.1 cm	56% (34/78)	81% (5/26)	2.83 (1.11–7.24)	0.0233
Spread_bulk_	50.3 cm	56% (27/62)	81% (8/42)	2.76 (1.25–6.08)	0.0086	50.3 cm	52% (30/62)	79% (9/42)	2.87 (1.36–6.05)	0.0038
Spread_patient_	366 cm	57% (26/60)	80% (9/44)	2.53 (1.19–5.41)	0.0128	366 cm	52% (29/60)	77% (10/44)	2.66 (1.30–5.47)	0.0056
MTV	332 ml	56% (25/57)	79% (10/47)	2.59 (1.24–5.39)	0.0085	332 ml	51% (28/57)	77% (11/47)	2.73 (1.36–5.49)	0.0033

“High”/”Low”: groups defined by the variable being more/less than the specified cutoff, HR (95%CI): Hazard ratio and corresponding 95% confidence interval based on Cox model, *p*-value: logrank test *p*-value, numbers between brackets represent the number of patients in a group who experienced an event over the total number of patients of the group; total number of patient is 104.

**FIGURE 4:**
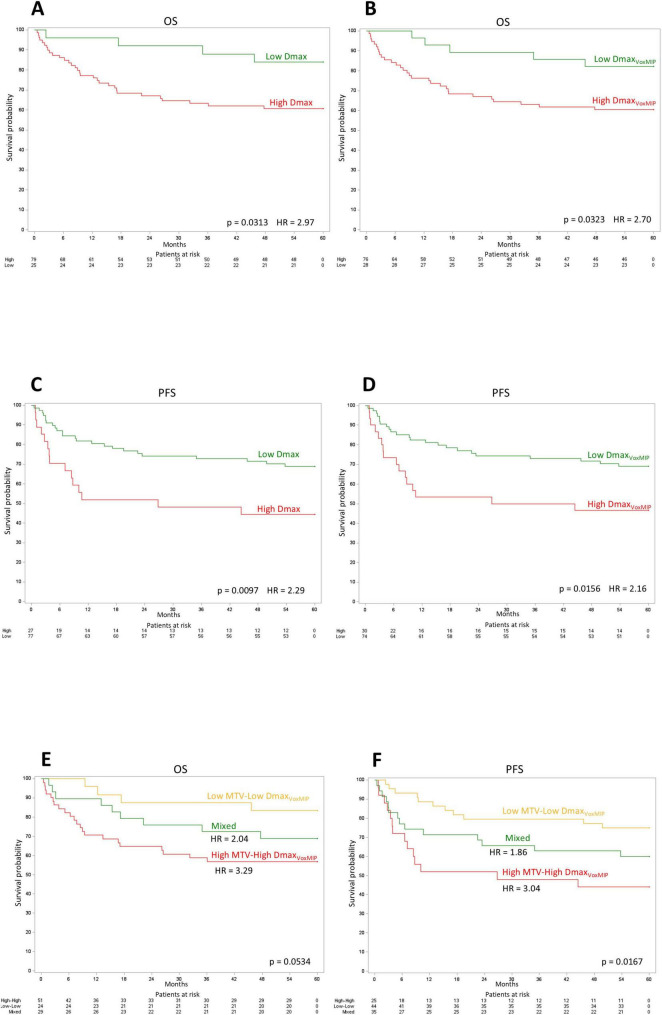
Kaplan-Meier curves. Kaplan-Meier OS curves for Dmax **(A)** and Dmax_VoxMIP_ **(B)**. Kaplan-Meier PFS curves for Dmax **(C)** and Dmax_VoxMIP_ **(D)**. Kaplan-Meier OS **(E)** and PFS **(F)** curves using a cross-classification of the MTV and Dmax_VoxMIP_ variables.

Kaplan-Meier curves of 5-year OS and PFS for Dmax and Dmax_VoxMIP_ according to the optimal dichotomization are shown in Figure 4. Based on a combination of Dmax_VoxMIP_ and MTV, three risk categories could be distinguished: group 1 with low Dmax_VoxMIP_ and low MTV (Low-Low), group 2 with either high Dmax_VoxMIP_ or high MTV (Mixed) and lastly group 3 with high Dmax_VoxMIP_ and high MTV (High-High). These three groups had nearly significantly different 5-y OS rates of 83, 69, and 57% respectively and significantly different 5-y PFS rates of 75, 60, and 44% (Figure 4). For groups 2 and 3, using group 1 as a reference, the 5-y OS HR were 2.04 and 3.29 and 5-y PFS HR were 1.86 and 3.04 (Figure 4).

The addition of MTV or DmaxVoxMIP (as continuous parameters) to survival models including the IPI score as a (categorical) explanatory variable for either overall survival or progression free survival endpoints, did not bring statistically significant improvement (Table 4). The absence of statistically significant improvement may be partly due to limited sample size, potentially affecting the power to detect incremental prognostic value of PET metrics.

## Discussion

In the present study, we showed in a retrospective bi-centric cohort, that the dissemination index Dmax_VoxMIP_, which is the distance between the outermost voxels of the most distant lymphoma sites, is a prognostic parameter with similar performance characteristics as Dmax.

First, confirming literature data, we found that Dmax, Dmax_Vox_, Dmax_bulk_, SPREAD_bulk_, SPREAD_patient_ carry significant prognostic value for 5-y OS and 5-y PFS (5–9). The method of Contal and Quigley (17) based on the logrank statistic that is best suited for survival analysis has been used (rather than the usual Youden criterion) to determine optimal cutoff values for these variables.

SDmax_Vox (Dmax_Vox_, calculated from the two most distant voxels and normalized by the BSA) has been shown to slightly improve the prognostic value of SDmax (calculated from the centroids) (5). This could potentially open the door to an easier method to gauge dissemination extent, by measuring the distance on a 2D MIP (5). Therefore, in this study, we introduced a new index of dissemination, measured manually on a 2D MIP in the coronal view: Dmax_VoxMIP_. The prognostic significance of Dmax_VoxMIP_ was demonstrated when patients were classified according to the optimal threshold determined by the Contal and O’Quigley method, showing statistically significant differences in 5-year OS and 5-year PFS. This provides additional evidence that advanced assessment of tumor spread is relevant in DLBCL patients (5–9).

It seems more intuitive to analyze lesion dissemination by considering the most distant points of the lesions rather than their center. Although Dmax and Dmax_Vox_ (or Dmax_VoxMIP_) are similar for small-sized lesions, a bulky lesion may lead to underestimation of the spread by Dmax. Furthermore, we noted that lesion selection can vary considerably depending on how the software segments the regions of interest (ROIs). This can be particularly important when lesions present unusual shapes and can wrongfully lead to the segmentation of a single lesion into several distinct regions. Manual confirmation of segmentation is always required for Dmax.

While Dmax depends on ROIs selection, Dmax_Vox_ and Dmax_VoxMIP_ are independent of it. Moreover, as their calculation does not rely on lesion centroids, both Dmax_Vox_ and Dmax_VoxMIP_ can be applied to patients with a single lesion, by measuring the longest intra-lesion diameter. Dmax_VoxMIP_ does not require any automated tumor segmentation program. On the other hand, Dmax_Vox_ and Dmax_VoxMIP_ are more sensitive to lesion edge definition than Dmax and may therefore be somewhat more influenced by image acquisition and reconstruction parameters.

The use of 2D MIP images was recently described in a study investigating whether MTV and Dmax could be replaced by surrogate parameters calculated automatically using an AI algorithm from just 2 MIPs (coronal and sagittal) (11). This study found that the delineation of lymphoma regions on 2D MIP images is faster than on 3D volumes, and that training an automated tumor segmentation algorithm is easier in 2D than in 3D. STMTV and SDmax were evaluated for their prognostic value in two independent cohorts of lymphoma patients (11). The results showed that STMTV and SDmax calculated automatically by AI have a strong prognostic value for progression-free survival and overall survival, comparable to that of TMTV and Dmax calculated from 3D volumes. The researchers also showed that using 2D MIP for parameter extraction reduced inter-expert variation in lesion delineation (11).

In our study, we used a more practical method with 2D MIP images, measuring the distance between the outermost voxels by hand. Dmax_VoxMIP_ showed a strong correlation with Dmax_Vox_ obtained using LIFEx software. The Bland-Altman plot revealed that the largest differences occurred for the largest distances, as expected. Additionally, the plot showed mostly positive differences, but also some negative ones, indicating that different endpoints were designated for Dmax_Vox_ and Dmax_VoxMIP_. Furthermore, we demonstrated that Dmax_VoxMIP_ is reproducible between observers. Compared with Dmax_Vox_, Dmax_VoxMIP_ yields comparable differences between patients alive or deceased at 5 years, or between patients with or without events within 5 years. It also provides similar overall and progression-free survival rates and risk stratification as Dmax. This method is simple, quick, and easily obtainable, making it a viable alternative for distance measurements.

A limitation of our method is the possible presence of one of the extreme lesions behind organs displaying high tracer concentrations such as the bladder or the heart. To exclude any lesion behind a physiological uptake in the heart or the bladder, the sagittal 2D MIP was also viewed.

We combined Dmax_VoxMIP_ to MTV to create three groups showing nearly significantly different 5-y OS rates and significantly different 5-y PFS rates similarly to what Cotterau et al. obtained in their articles (6, 8). However, combining PET-derived parameters (dissemination and MTV) showed no improvement to IPI’s predictive value. This can most likely be explained by the fact that the prognostic information of these PET metrics is already covered by constituents of the IPI score (18–20).

A limitation of this work is the use of a retrospective design, and the disadvantages that entails, such as misclassification bias (e.g., patients being assigned to the wrong Ann Arbor stage), patients lost to follow-up, missing data, etc. Moreover, cut-offs were obtained from this study dataset. Therefore, they are only valid for this specific cohort, as is the case for most studies on dissemination features (10).

## Conclusion

To conclude, Dmax_VoxMIP_ is an easy parameter to measure on [^18^F]FDG PET/CT, foregoing the use of a segmentation program. If dissemination parameters prove useful clinically, Dmax could possibly be advantageously replaced by Dmax_VoxMIP_, but future prospective studies are needed to confirm our results.

## Data Availability

The raw data supporting the conclusions of this article will be made available by the authors, without undue reservation.

## Supplementary material

Supplementary Figure 1 Bland-Altman plot comparing repeated Dmax_VoxMIP_ measurements by one observer.

Supplementary Figure 2 Bland-Altman plot comparing Dmax_VoxMIP_ measurements between observers 2 and 3.
