# Supplementary figures and images for: Prognostic value of a simple distance index derived from PET maximum intensity projection
Source: Front Med (Lausanne). 2025 Jul 14;12:1565525. doi: 10.3389/fmed.2025.1565525 (PMC12301333; doi:10.3389/fmed.2025.1565525)

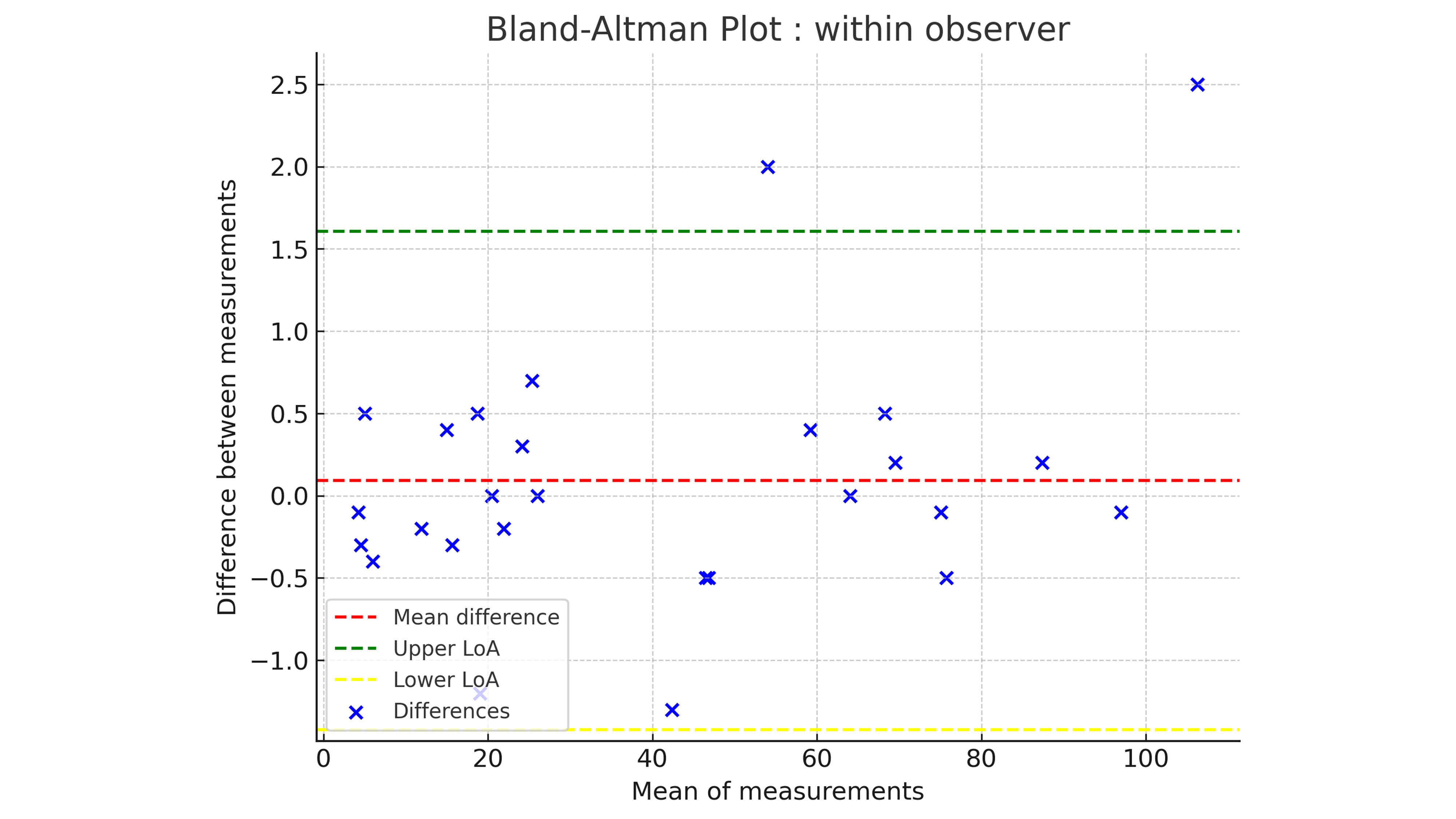

Supplement: Supplementary Figure 1 — Bland-Altman plot comparing repeated DmaxVoxMIP measurements by one observer. [file Image_1.tif]

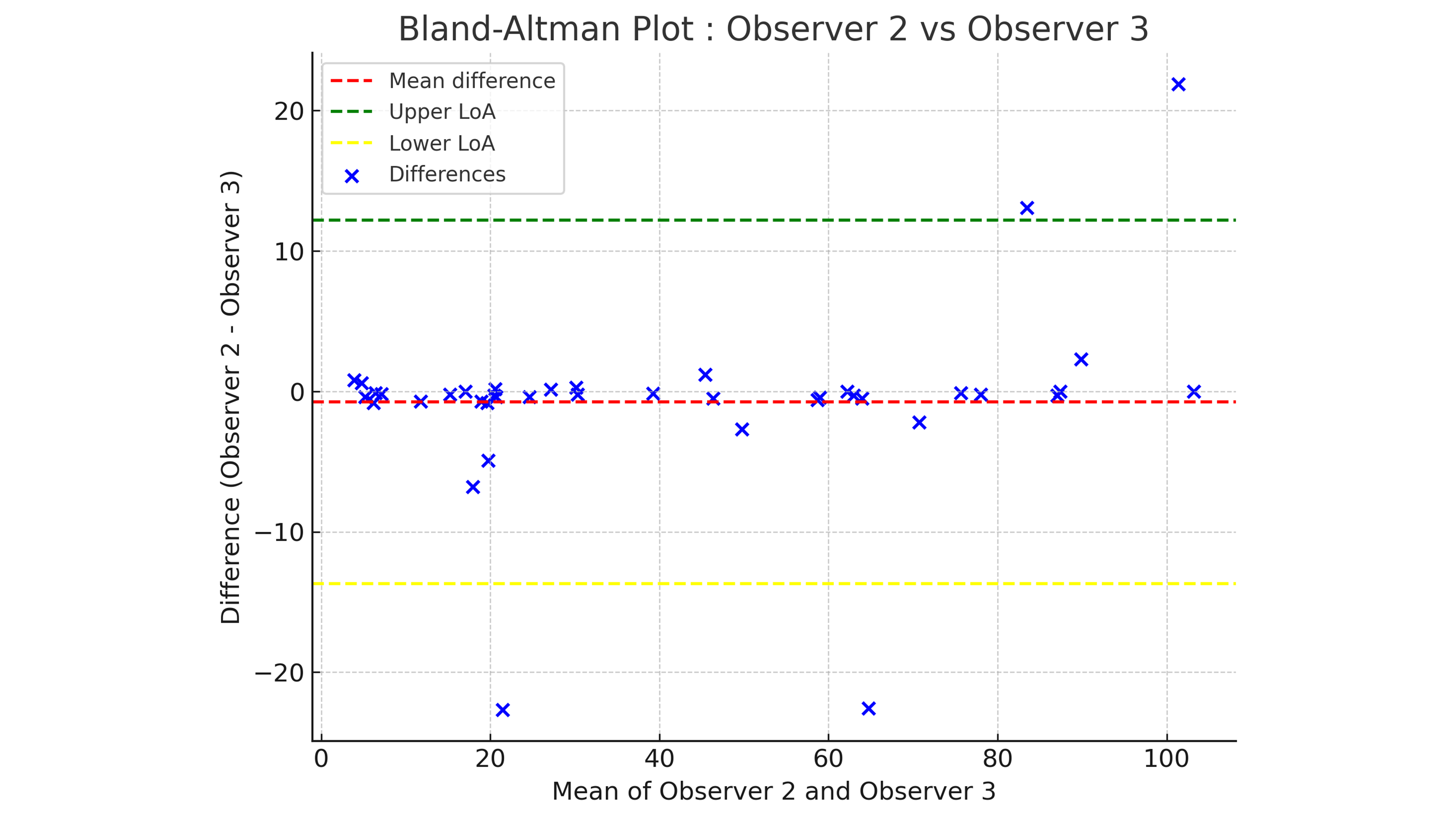

Supplement: Supplementary Figure 2 — Bland-Altman plot comparing DmaxVoxMIP measurements between observers 2 and 3. [file Image_2.tif]
